# The Means by Which the Temperature of the Body Is Maintained in Health and Disease

**Published:** 1897-06-26

**Authors:** W. Hale White

**Affiliations:** Physician to, and Lecturer on Pharmacology at, Guy's Hospital


### Medical Progress and Hospital Clinics.

[*The Editor will be glad to receive offers of co-operation and contributions from members of the profession. All letters should be addressed to* The Editor, at the Office, 28 & 29, Southampton Street, Strand, London, W.C.]

### THE MEANS BY WHICH THE TEMPERATURE OF THE BODY IS MAINTAINED IN HEALTH AND DISEASE.

The Croonian Lectures, delivered before the Royal College of Physicians of London.

By W. Hale White, M.D., F.R.C.P. Lond., Physician to, and Lecturer on Pharmacology at, Guy's Hospital.

### Lecture II.

It was shown in the last lecture that it would be possible by taking simultaneous observations of the internal temperature, the surface temperature, and the amount of sweat secreted, to find out whether a rise of internal temperature was due to a diminution in the loss of heat or an increase in its production. On this basis Dr. Hale White had made various experiments. After remaining for 14 minutes in a bath, which in that time cooled from 112 to 108 deg. F., the mouth temperature was found (after drying and lying down in a dressing gown) to be 103·2 F.

The sweating, however, was very profuse, and in the course of half an hour the temperature had fallen to normal. A second bath produced variations in the same directions, but not so marked in degree.

These experiments showed what an important influence free diaphoresis and evaporation of the sweat had in reducing temperature which had been artificially raised, for it had to be noted that during this free sweating the surface temperature was so much lowered that notwithstanding the high internal temperature the loss of heat by radiation and conduction must have been actually less than normal. The effect of a Turkish bath was much the same; the sweating was profuse, even after removal from the bath.

Experiments on rabbits and observations on cases of cerebral hæmorrhage in man where the corpus striatum had been damaged showed that this injury caused a rise of internal temperature on the paralysed side. Not only so, but in one case the sweating was so much greater on the paralysed than on the sound side that the surface temperatures were equal, while in other cases, notwithstanding the loss of heat from free sweating and evaporation, even the surface temperature on the paralysed side remained the higher. The quantity of heat produced must then have been considerably greater in the paralysed arm than in the other. It was not a mere matter of greater quantity of blood, but of increase in the amount of heat produced by metabolism in the tissues.

Coming, then, to the question of heat production and heat loss in the several forms of "fever," many observations were recorded which tended to show on the one hand that the pyrexia in such conditions was the resultant, as indeed was the case with the normal temperature of the body, of these two factors, and on the other that the degree to which the one or the other (production or loss) was involved varied much in the different forms of fever, so that the resultant internal temperature was far from being a correct index of the tissue changes going on.

In typhoid fever the amount of sweat secreted was in all cases small—less than when the temperature had subsided. The surface temperature also rose less than the internal temperature. Thus the heat loss—whether by evaporation, radiation, or conduction—was not increased proportionately to the rise of internal temperature during the fever. There was then distinct evidence that in typhoid fever the loss of heat was diminished in proportion to its production, and this diminished loss no doubt helped to raise the internal temperature, although probably increased production was also concerned. Thus perhaps was to be explained the fact that although in typhoid fever the temperature remained high for a long time, the mortality was low, for it was obvious that it was more economical of energy to raise the temperature by diminishing the loss of heat than by increasing the production.

In pneumonia the surface temperature was high during the pyrexia, and probably the sweat was not much less than the normal, therefore the pyrexia was for the most part due to increased production. It was important to note the greatly increased heat loss from excessive sweating which took place in this disease in connection with the crisis, and lasted for some days after it.

In erysipelas the increased heat was shown by the same criteria to be due entirely to increased production.

In certain cases of suppuration the rise of surface temperature was proportionately less than that of the internal temperature, which latter fell with the occurrence of free perspiration, so that in part at least the pyrexia was due to diminished loss of heat.

A very interesting observation made by Maragliano was referred to as showing how very much the temperature of some fevers depended upon the state of the cutaneous circulation, or, in other words, on loss from radiation and conduction. He observed that in the fevers he was investigating the volume of the arm, the variation of which is chiefly dependent on the amount of blood circulating in the vessels of the skin, diminished as the temperature rose, *i.e.*, that there was a lessened loss by radiation and conduction at that time, the contraction of the vessels apparently preceding the rise of temperature.

Not only, however, was the cause of pyrexia differ in different fevers, but the manner and degree in which people threw off heat and thus avoided pyrexia differed in different individuals. A series of observations which Dr. Hale White and Dr. Archibald Garrod had made upon each other were detailed, by which it was shown that they accommodated differently, the one always having a higher surface temperature, as well as more perspiration, and thus in every way losing heat faster than the other, and therefore requiring to produce a considerably larger quantity of heat, as measured in calories, to maintain the same internal temperature.

### PELVIC HÆMATOCELE.

First of all, what do we mean by a pelvic hæmatocele? Secondly, how is it caused? Lastly, how is it to be treated? In an address recently delivered before the Nottingham Medico-Chirurgical Society, Dr. Cullingworth answers these questions very clearly. A pelvic hæmatocele he defines as an intra-peritoneal effusion of blood in the pelvis, usually situated behind the uterus and displacing it, and limited towards the general cavity of the peritoneum by encapsulisation or adhesions. He does not include in the term unlimited effusions of blood into the general peritoneal cavity, on the one hand, nor effusions in the connective tissue of the broad ligament, on the other.

It has to be recognised, then, that in this limited sense it is generally agreed that, in at least 19 cases out of every 20, the effusion, when not traumatic, is in one way or another the result of tubal gestation. It is important, then, to consider what may happen when the ovum instead of passing on into the uterus is detained within the tube. In this position the vascular connection between the ovum and the maternal structures is always more or less insufficient; there is no properly constituted decidua, and the muscular coats of the tube stretch, instead of hypertrophying as those of the uterus do. Hence the liability to premature death of the ovum, to hæmorrhage in and around the membranes and to rupture of the tube, the site of rupture being the spot where the penetration of the chorionic villi have made the walls especially weak. This rupture may take place directly into the peritoneum or into the connective tissue between the two peritoneal coverings of the broad ligament, in the latter case giving rise to a hæmatoma of the broad ligament, where the ovum may die, or may develop, and ultimately by a secondary rupture escape into the peritoneum. The point which Dr. Cullingworth insists upon is that rupture of the tube into the peritoneum, whether primary or secondary, tends in by far the majority of cases to produce a free unlimited effusion rather than a hæmatocele. The reason of this is easily understood. Free effusion in the peritoneal cavity depends chiefly on extensive and especially on the rapid hæmorrhage, hence it commonly occurs with rupture. What, then, is the cause of that slow bleeding which tends especially to produce hæmatocele? To this Dr. Cullingworth answers that those recurring hæmorrhages by which what is called a tubal mole is caused, if they take place before the abdominal ostium of the tube is closed, tend not only to distend the tube with clot but to flow out into the pelvis, and thus to produce the condition here in question, viz., pelvic hæmatocele. Along with the blood the ovum may escape, or the embryo alone may reach the peritoneum, or the whole ovum may remain in the tube and blood alone may find its way out; but in any case the hæmorrhage is generally not excessive or poured out in a rapid stream; it is most frequently a continued trickle. This is just the condition which is most favourable to the production of an encapsuled hæmatocele, and Dr. Cullingworth urges that this latter condition is not usually the result of the rupture of a tubal gestation, as is commonly stated in the text books, but that it is far more frequently the result of hæmorrhage from the open abdominal ostium of a pregnant but unruptured tube; and that it is only in very exceptional cases of rupture that the blood is poured out in sufficiently small amount, and with the requisite deliberation, to result in the formation of a hæmatocele.

Now this is a matter of some practical importance in regard to treatment. As Bland Sutton has shown in most cases of tubal gestation, the abdominal ostium of the pregnant tube becomes occluded from the sixth to the eighth week. It is then during the early weeks of a tubal pregnancy that effusion of blood into the peritoneum without rupture of the tube is most likely to take place, and, on the other hand, an effusion occurring after that time is more likely to be due to either primary or secondary rupture of the tube. So long as we believe that all pelvic hæmatoceles are due to rupture of the tube we are met with the difficulty that there is nothing to explain why some cases of rupture should lead to such fatal consequences, while other cases if let alone tend to recover themselves. But if we recognise that the hæmatocele which recovers is due to a different cause from the free effusion, for the treatment of which operation is imperatively necessary, we see at once that what we have to do in the first instance is to come to a conclusion as to whether the case is one of rupture of the tube or one of effusion at its open extremity. If there is reason to believe that the case is one of rupture of the tube, the chances are very great that the hæmorrhage will be free in the peritoneal cavity, and when this is the case the necessity for immediate operation, if the condition of the patient permits, is indisputable. If, on the other hand, we can believe that the bleeding has come from an open tube and is likely to become encapsuled, we may wait, unless the swelling is found to be increasing, or when, from the repeated occurrence of attacks of pain and faintness, there is reason to believe that hæmorrhage is still going on, in which case, again, operation is imperative. Here, then, comes in the importance of the time of occurrence, the period at which the first sign of hæmorrhage appears. It can only be when the clinical history points unmistakeably to the hæmorrhage having occurred within the early weeks of pregnancy that we can safely count upon its being likely to be limited in the form of a pelvic hæmatocele. At any later stage the embryo and other products are too large, and the risk of further hæmorrhage is too considerable for non-operative treatment to be justifiable. Whenever, also, the severity of the initial symptoms is sufficient to lead to a suspicion that the case is one of rupture, even should it occur quite early, there should be no hesitation in advising operation. This is a neat and crisp conclusion, and will be of considerable help in many doubtful cases.

